# Rituximab for pulmonary lymphomatoid granulomatosis which developed as a complication of methotrexate and azathioprine therapy for rheumatoid arthritis

**DOI:** 10.1186/2193-1801-3-751

**Published:** 2014-12-18

**Authors:** Athar Barakat, Karan Grover, Rohit Peshin

**Affiliations:** Department of Rheumatology, Nobles Hospital, Isle of Man, IM44RJ UK

**Keywords:** Pulmonary Lymphomatoid granulomatosis, Rheumatoid arthritis, Rituximab

## Abstract

We report the case of a patient with Rheumatoid Arthritis [RA] presenting with clinical-pathological and radiological features of Pulmonary Lymphomatoid Granulomatosis (PLG). This is a rare lung disorder characterized by multiple nodular lesions with lymphocytic invasion of vascular walls. We present one such case of PLG secondary to Methotrexate and Azathioprine therapy, who was successfully treated with Steroids and Rituximab. We wish to highlight the importance of lung biopsy in the diagnosis and the use of rituximab as a treatment modality for RA as well as PLG.

**Electronic supplementary material:**

The online version of this article (doi:10.1186/2193-1801-3-751) contains supplementary material, which is available to authorized users.

## Introduction

We report the case of a patient with Rheumatoid Arthritis [RA] presenting with clinical-pathological and radiological features of Pulmonary Lymphomatoid Granulomatosis (PLG). This is a rare lung disorder characterized by multiple nodular lesions with lymphocytic invasion of vascular walls. We present one such case of PLG secondary to Methotrexate and Azathioprine therapy, who was successfully treated with Steroids and Rituximab. We wish to highlight the importance of lung biopsy in the diagnosis and the use of rituximab as a treatment modality for RA as well as PLG.

## Case report

A 60 year old lady with anti-CCP and sero-positive rheumatoid arthritis [RA] on methotrexate (MTX, 20 mg once weekly), hydroxycholroquine (HXQ, 200 mg twice daily), azathioprine (AZA, 50 mg twice daily), presented with a history of weight loss (2 stone in 6 months), poor appetite for 5 months, non-productive cough and non exertional shortness of breath of few days duration.

Examination was unremarkable except for mouth ulcers, an erythematous rash with irregular borders above the upper lip and on trunk. There was no focal neurology. White cell count was elevated along with a predominantly raised neutrophil count, and CRP was elevated too. Biochemical profile was unremarkable.

Chest x-ray revealed increased coarse, linear shadowing in the mid- and lower zones, prominent hila, bilateral Kerley B lines, and a pleural reaction on the left. Nodular shadows were present bilaterally, the largest measuring 18 mm.

Methotrexate and Azathioprine were discontinued. As concerns were raised about methotrexate lung disease, Folinic acid rescue was instituted and broad spectrum antibiotic cover with IV levofloxacin and oral clarithromycin therapy was commenced.

**Figure 1:**
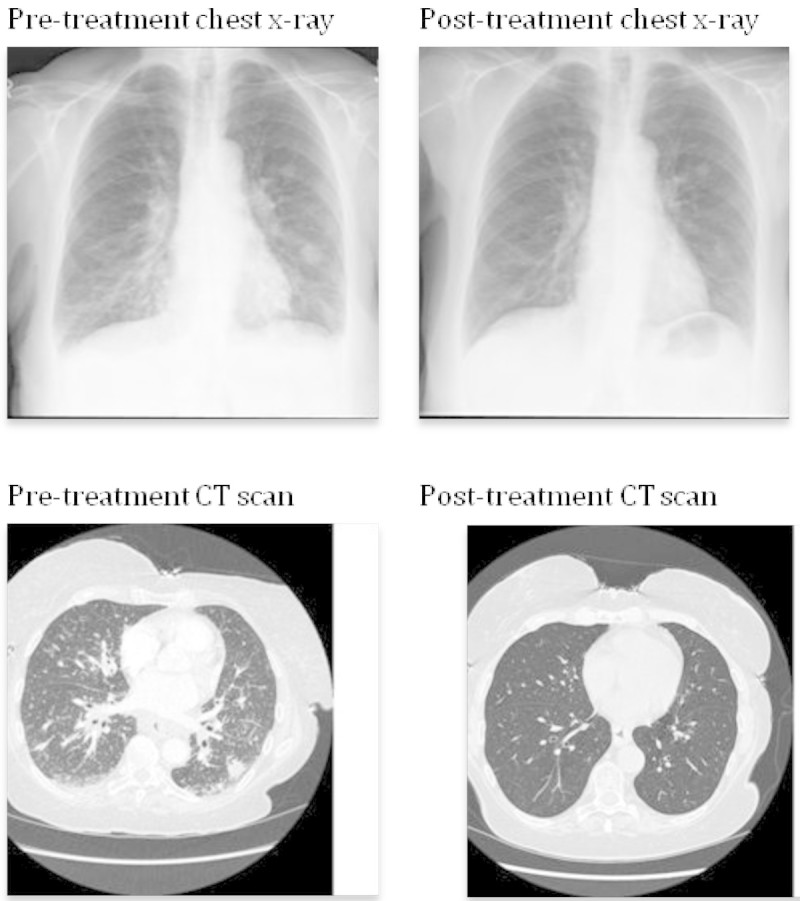
**CT scan chest.**

CT scan revealed mediastinal (complex confluent subcarinal lymph node mass measuring 5.6 cm), aortopulmonary, paratracheal, right tracheobronchial, axillary, upper abdominal (1.5 cm), external ileac and right inguinal lymphadenopathy (largest 10.7 mm). There was widespread reticulonodular shadowing, subpleural consolidation and congestion of interlobular lymphatics. A small right pleural effusion and little pleural thickening/pleural reaction was reported in dependent parts of the left lower lobe (Figure 1).

Lung function tests showed reduced diffusion (TLCO 62% of predicted), with no evidence of restrictive ventilatory defect.

Our Differential included lymphoma, sarcoidosis, Wegener’s, pulmonary fibrosis and malignancy.

Lung biopsy conclusively diagnosed Pulmonary Lymphomatoid Granulomatosis [PLG]. EBV PCR was negative.

An opinion was sought from the haematologists who confirmed the diagnosis, malignancy was ruled out, and as the lung picture had improved considerably with steroids, it was agreed in an MDT setting that Rituximab could be used to treat both the RA and PLG.

The patient improved significantly on steroids (Figure 1) as well as rituximab infusions [dose 375 mg/m2 for 4 weeks]. The clinical response to treatment was very good, and a post-treatment CT chest shows complete resolution of lung changes.

The patient has been under surveillance for more than a year now with no recurrence of joint symptoms, lung complications or any extra articular complication. She has a very good quality of life now. The rituximab therapy really brought the disease as well as the PLG into remission.

## Discussion

Pulmonary lymphomatoid granulomatosis (PLG) is a rare lung disorder characterized by multiple nodular lesions with lymphocytic invasion of vascular walls.

It is a nonspecific manifestation of conditions, including autoimmunity, immunodeficiency, infection, malignancy, lymphoproliferative disorder (Pittaluga et al. 2008; Katzenstein et al. 1979; Sordillo et al. 1982), can be secondary to immunosuppressive medications such as azathioprine, methotrexate, and imatinib where the disorder resolves after discontinuing medications (Katherine Martin et al. 2009; Kameda et al. 2007; Pfistershammer et al. 2010; Pisani and DeRemee 1990). It generally presents between 30 and 50 years, but any age group can be affected (Koss et al. 1986). It has been associated with Epstein-Barr virus infection in most cases (Pittaluga et al. 2008). Immune defects may lead to an abnormal host response to EBV infection (Pittaluga et al. 2008; Jaffe and Wilson 1997).

Lungs are most commonly involved (>90 percent) (Pittaluga et al. 2008) with or without skin and neurologic involvement. The commonest presenting picture includes fever, cough, rash/nodules, malaise, weight loss, neurologic abnormalities, dyspnoea, and chest pain (Katzenstein et al. 1979).

Laboratory investigations are nonspecific, but pulmonary function test abnormalities can be characteristic (Katzenstein et al. 1979).

Chest radiography typically reveals multiple poorly defined nodules and/or masses in the mid- and lower-lung zones with possible diffuse reticular abnormalities (Jaffe and Wilson 1997; Katzenstein et al. 1979). Diagnosis requires the histopathologic triad of polymorphic lymphoid infiltrates, transmural infiltration of arteries and veins by lymphoid cells, and necrotic foci (not typical granulomas) within the lymphoid infiltrates (Pittaluga et al. 2008).

The prognosis varies (Katzenstein et al. 1979) from remission without treatment (Katzenstein et al. 1979; Jaffe and Wilson 1997) to death within 2 years from malignant lymphoma.

Treatment differs according to symptoms, use of medication associated with PLG, extent of extrapulmonary involvement, and histopathologic grade. Medication implicated in the disorder should be stopped with repeated imaging over weeks to months (Pittaluga et al. 2008).

To our knowledge after literature search, this is the first case report of the use of Rituximab in treatment of PLG which had developed as a complication of DMARD therapy in a patient with RA.

## Key message

Lymphatoid granulomatosis is a rare lymphoproliferative disorder primarily affecting the lung, with variable clinical outcome.

## Consent

Written informed consent was obtained from the patient for the publication of this report and any accompanying images.

## Authors’ original submitted files for images

Below are the links to the authors’ original submitted files for images. Authors’ original file for figure 1
