# Supplementary material for: Rituximab for pulmonary lymphomatoid granulomatosis which developed as a complication of methotrexate and azathioprine therapy for rheumatoid arthritis
Source: Springerplus. 2014 Dec 18;3:751. doi: 10.1186/2193-1801-3-751 (PMC4320142; doi:10.1186/2193-1801-3-751)

Pre-treatment chest x-ray

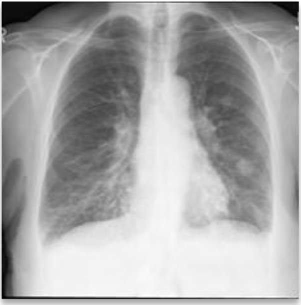

Post-treatment chest x-ray

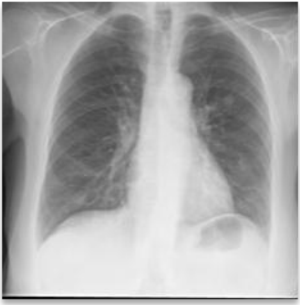

Pre-treatment CT scan

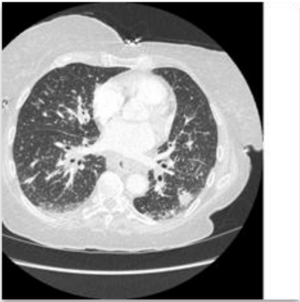

Post-treatment CT scan

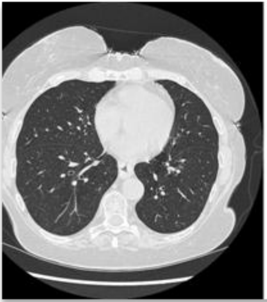

Supplement: Supplementary file 1 — Authors’ original file for figure 1 [file 40064_2014_1525_MOESM1_ESM.pdf]
